# Bridging the attitude-behaviour gap: An explanation of travel mode choice using analytical sociology

**DOI:** 10.1371/journal.pone.0330073

**Published:** 2025-10-15

**Authors:** Johannes Weyer, Sebastian Hoffmann

**Affiliations:** Department of Social Sciences, TU Dortmund University, Dortmund, Germany; Khalifa University, UNITED ARAB EMIRATES

## Abstract

The aim of this work is to improve the explanatory power of models of transport mode choice and thus contribute to the mobility transition. The authors develop a new model of mobility behaviour called xMooBe: It incorporates elements from attitudinal and choice models and combines them with a sociological theory of action, which has its roots in analytical sociology. xMooBe is based on a simple model of decision-making (with a manageable number of variables) and expands it by taking into account additional contextual factors such as car ownership and public transport availability. The study uses a mixed-methods approach that combines statistical analysis of survey data (including regression analysis), theory-based modelling of (bounded-rational) everyday decision making and thought experiments to identify options for behavioural change. Instead of relying on manifest statements of behavioural intentions, xMooBe applies an extended version of the subjective expected utility theory, which refers to latent preferences and subjective perceptions (plus contextual factors). The mixed-methods approach was used to validate xMooBe and to test different assumptions about (policy) measures that could influence transport mode choice in terms of sustainability. xMooBe achieves up to 80 percent accuracy in explaining behaviour – and thus differs from many other studies with partly inconsistent results. xMooBe helps to understand why people behave in ways that are inconsistent with their attitudes, e.g., in the case of car-using cyclists, and thus helps to bridge the gap between attitude and behaviour. In most cases, known contextual factors (such as car ownership, state of the cycle network, etc.) help to explain this gap. At the same time, they serve as a starting point for interventions whose potential impact has been tested through experimentation.

## 1. Introduction

Mobility researchers in the social sciences and related fields agree that people’s mobility behaviour can be explained by several individual, social, and structural factors such as personal attitudes, social norms, transport infrastructure, et cetera. Concepts that use statistical analysis to identify determinants of mobility behaviour are widespread, for example through correlations between bundles of variables, regression analysis or structural equation modelling. This has resulted in a variety of methodologies and models, some of which are very complex and provide rather mixed or even inconsistent results as some review studies report (cf. [1]). This presents a challenge, since motorized private transportation makes up a large share of pollutant emissions [2]. Gaining a deeper understanding of the mechanisms that shape (un-)sustainable transport mode choices is thus crucial in finding (political) ways to support behavioural changes.

The best-known concepts for investigating mobility behaviour are attitude-based models and choice models, which are sometimes combined as hybrid choice models. Both have their strengths, but also some weaknesses, which has led us to rethink urban mobility by incorporating insights from sociological theory of action and create the Extended Model of Mobility Behaviour (xMooBe).

xMooBe is a sociological model of mobility behaviour, combining concepts from attitude-related models and choice models. It addresses the subjective-rational process of decision-making in the case of transport mode choice and is deliberately designed as a simple model that entails only a limited, manageable set of variables. xMooBe makes it possible to test different interventions for triggering behavioural change and to derive policy recommendations that differ from those of most existing studies.

### 1.1. Outlook of the paper

After providing a brief overview of common models of transport mode choice in Sections 1.1 to 1.2, we derive and propose an Extended Model of Mobility Behaviour (xMooBe, Section 2). It extends the established concept of subjective expected utility (SEU), which is based on individual preferences and subjective perceptions (Section 2.1), by including contextual factors, such as car ownership, distance to work and availability of public transport services (Section 2.2). Using the dataset from a large-scale mobility survey of university members in the Ruhr area (Sections 2.4), xMooBe was empirically tested (Sections 3 and 4).

With this extended model, a match of up to 80 percent between predicted and actual behaviour can be achieved, which might help to bridge the attitude-behaviour gap. The model not only helps to identify the factors that shape people’s daily mobility, but also shows starting points for behavioural changes towards sustainable mobility.

### 1.2. Attitude-related models

Most prominent in sociological or psychological studies of travel behaviour are concepts referring to the Theory of Planned Behaviour (TPB) or – in the case of acceptance of new mobility services – to the Technology Acceptance Model (TAM).

**Fig 1:**
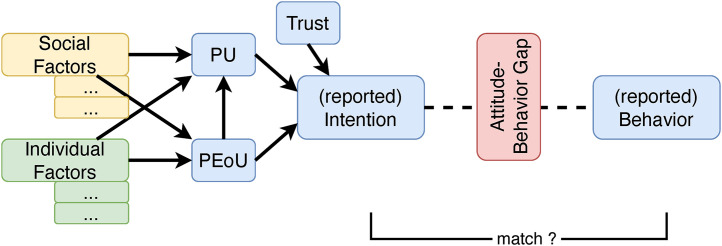
General scheme of attitude-related models like TPB and TAM (own presentation, based on various sources).

Despite different objectives and concepts in detail, studies adhering this approach point at various individual and social factors. These affect the dependent variable “behavioural intention” either directly (TPB) or indirectly via two intermediate variables (TAM): perceived usefulness (PU) and perceived ease of use (PEoU), and – more recently – also trust [3,4], as depicted in the general scheme in Fig 1.

However, researchers do not agree on the number of external factors and their importance; instead, research has continuously focused on finding new variables – many of them with minor effect strengths [5] – or new model variants [6]. More elaborated models such as TAM 2 or TAM 3 have also extended the number and the scope of individual or social variables, which led to complaints about a confusing variety of factors [7]. Although integrated models, like the UTAUT, have been developed in the area of technology acceptance, this is not the case for more general mobility behaviour and transport mode choice. Here, an integrated and comprehensive model is still missing, which includes all relevant factors as well as their complex interactions, and thus contributes to a better understanding of the mechanisms that shape and guide individual behaviour.

Additionally, many attitude-related models rely on self-reported behavioural *intentions* of respondents, who are confronted with only one particular action alternative, e.g., using active modes of transport more frequently [7], instead of various alternatives as in the case of choice models [8]. Furthermore, many questionnaires ask directly for manifest attitudes towards a specific behaviour, e.g., using the car [9], instead of indirectly referring to latent, mode-unspecific preferences.

Only few studies also take into account the actual behaviour, e.g., the (self-reported) usage of various modes of transport on a typical day of the week, and address the match of intentions and behaviour [7]. Concerning this relationship, there is an ongoing debate pointing at an attitude-behaviour gap (cf. vertical box in Fig 1) – a discrepancy between attitudes, e.g., towards environmental protection, and actual behaviour, e.g., buying or driving cars with internal combustion engines [10,11]. Studies on cognitive dissonance also give evidence that many people use a non-preferred mode of transport. For example, “bike-lovers” who still travel by train or by car [12] challenge basic assumptions of attitude-related models. Recently, Borriello and Rose [13] approached the attitude-behaviour gap by exploring the role of different kinds of attitudes: memory-based, long-term, *global* attitudes that are not connected to a specific behaviour (e.g., eco-friendliness), and more situational, on-the-spot, *localized* attitudes that do refer to a specific behaviour (e.g., recycling). They conclude that both attitudes need to be considered, since both have a significant influence on individual choices and the omission of one (i.e., localized) may lead to inconsistent estimates (ibid.: 162).

Although many TPB studies argue that attitudes influence behaviour and behavioural change will start with adjusting attitudes, evidence on this causation is weak. Empirical studies even suggest that behaviour (e.g., regularly using public transport) affects attitudes towards this particular behaviour much stronger than vice versa [14]. However, even those critical studies do not suggest reasons, why attitudes and behaviour frequently do not match – a matter that is not only important to scientists, but also to policy makers.

Furthermore, attitude-related models do not intend to investigate the everyday, partly routinized process of choosing between various modes of transport, which remains a black box (e.g., [9]). Hence, the individuality of heterogeneous people making autonomous decisions disappears in the statistical calculations (e.g., [15]). However, depending on their subjective needs and their subjective views of the world, people may decide differently (and not always perfectly rational), when confronted with similar situations – a puzzle that sociologists might be interested in solving.

Hence, the results of attitude-related studies often are “mixed”, as Scheiner and Holz-Rau report [6]. Similarly, after having identified more than 60 factors, Javaid et al. conclude in their review of reviews that “all three dimensions [i.e., individual, social, and other factors] unambiguously interfere with mode choice” [5].

Finally, it seems to be difficult to derive recommendations for policy makers based on attitude-related analysis. Many studies argue that politics should improve conditions so that people change their attitudes and, finally, their behaviour [12,15], while others point at the methodological problems of this claim [16]. In other cases, rather general recommendations are given such as politics could “initiate behaviour nudging through pilot projects” [15].

### 1.3. (Hybrid) Choice models

In other disciplines, such as transport economy or sociology, but also in agent-based modelling of urban transportation, choice models are more prominent [17,18]. These models mostly consider (travel) time and (travel) costs as two main factors affecting mode choice, which can be mathematically modelled, mostly assuming a rational mode of decision-making [19,20]. The purpose of this approach is to determine travel demand, which is expressed by the probability that a certain number of people will use a specific transport mode in the future, based on behavioural data from the past.

The most important feature, compared to attitude-related models, is the calculation of the utility of *several* transport modes, which can be mathematically deduced from attributes of the respective mode (travel time, costs etc.) and of individual characteristics (age, gender, income etc.). According to the standard assumption of rational choice models, individuals choose the option with their highest individual utility in order to satisfy their needs.

Standard rational choice models assume that all human beings make equally perfect rational decisions, which is an unrealistic assumption given the heterogeneity of individuals and the variety of their choices. The usual way to cope with the ‘problem’ of human individuality is to assume that there are unobserved ‘blind spots’ on part of the decision-maker or the decision-making process. This may encompass factors like fluctuations in attribute-related preferences (among multiple decision-makers as well as individually over time), errors in the evaluation of attributes, or incomplete information about relevant attributes (on part of the researcher). These factors are represented mathematically by some degree of randomness in the utility function (“random utility theory”, cf. [18]).

Although choice models are useful tools for calculating travel demand (with sophisticated statistics), the non-consideration of human factors is a serious disadvantage, especially if behavioural change is investigated. Hence, various proposals have been made to combine models from psychology and economy, e.g., in the form of a Decision-Theoretic Model of behaviour change (DTM). It claims that attitude-related models could be complemented by utility-driven choices, while choice-based models should be extended by including individual and social factors ([8], see also [21]).

**Fig 2:**
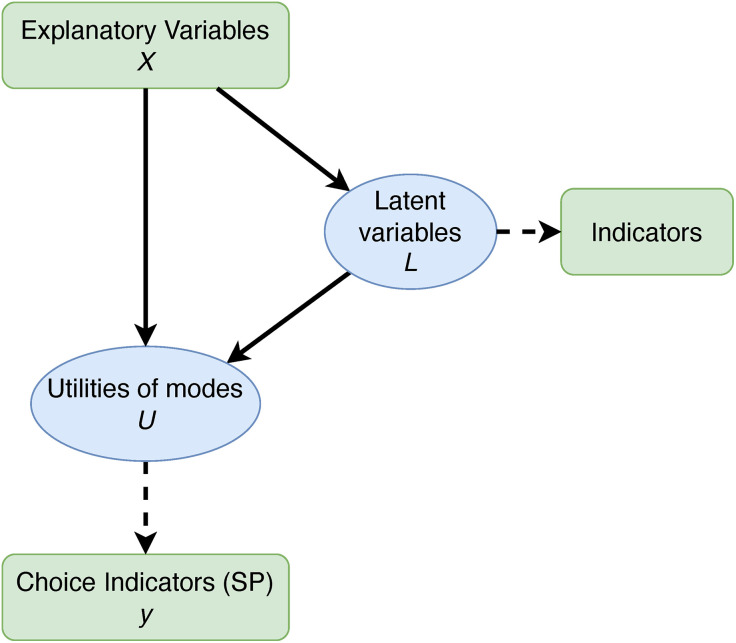
Hybrid choice models (adapted from [22]).

Hybrid choice models (cf. Fig 2) also incorporate individual attitudes and preferences as explanatory variables. Namely, they are modelled as additional latent variables that cannot be directly observed, but are supposed to distinctly affect the result of utility calculations [23–25]. Despite these improvements, choice models suffer from relying on the explicitly stated preferences (SP) of respondents, e.g., concerning their preferences towards using a *specific* transport mode (e.g., [25,22]). This frequently leads to unsurprising results such as: “willingness to walk and to cycle has a positive effect on the choice of those alternatives” [22] or “stress decreases satisfaction” [25].

Choice models are capable of describing the current state of the mobility system but lack a deeper explanation of the motives that drive people to act as they do. Decisions are modelled referring to well-known behavioural patterns, relying on past statistical data. However, this may involve the risk of drawing conclusions about future behaviour from past patterns (cf. [16]), without regarding the underlying, mode-unspecific motives of people, that may entail more options for changing behaviour than stated preferences reveal. Hence, a general (sociological) theory of action, which better accounts for the individuality of people, might help to improve those choice models (see next Section).

## 2. Concepts and methods

### 2.1. Sociological model of decision-making

There are only a few attempts to model people’s mobility-related actions based on a general theory of action and consequently to approach the question of whether and how a change in mobility behaviour might be possible [26,27]. While a few of them have already been applied in empirical studies on mobility behaviour (cf. [28]), others remain in a conceptual state.

**Fig 3:**
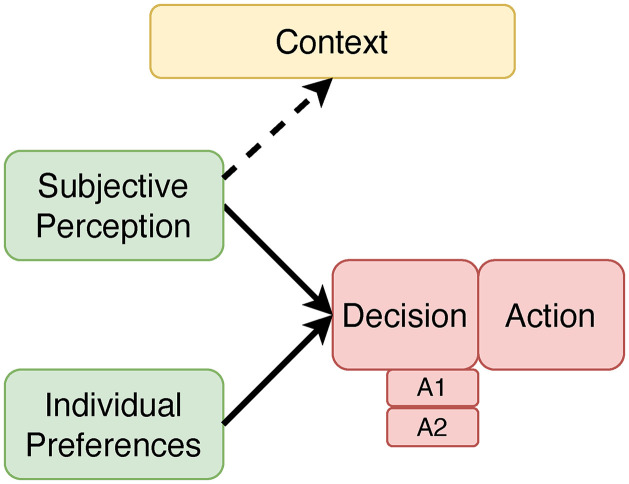
Standard model of decision making (adapted from [29]).

Analytical sociologists have developed a basic, versatile model of decision-making that is applicable to various settings. This model reconstructs and explains the everyday behaviour of human beings, taking into account their individual preferences (i.e., needs and objectives), as well as subjective perceptions about their situation and context (cf. Fig 3). People’s decisions are explained as the result of a choice between different action alternatives (A1, A2, …), assuming that they usually select the alternative that – from a subjective point of view – best satisfies their own needs [30–34]. These benefits do not have to be exclusively monetary; other factors, such as convenience or recognition by other people, may also play a role.

While initially similar to standard (rational) choice models, researchers from analytical sociology emphasize and systematically consider the subjectivity, individuality, situativity, and bounded rationality of human action.

This analytical model does not claim that people permanently make conscious choices, but that even their everyday routines can be reconstructed and finally explained in terms of utility-maximization. The subjective expected utility (SEU) is based on two factors:

- *Individual* preferences: the general importance attributed to the achievement of desired objectives (e.g., travelling quickly, environmentally friendly, comfortably, safely, reliably, or cost-effectively);
- The *subjective* definition of the situation: the perceived probability of achieving a desired objective (e.g., travelling fast) by selecting a specific action among multiple alternatives (e.g., car or bicycle).

The latter variable is a major component of the sociological model of decision-making, which surprisingly is not taken into account in other models that rely on preferences only.

The total subjective expected utility (SEU) of each available action alternative (A) is calculated as the summed product of the objectives (O), which are weighted by individual preferences (U, “utility”), and the subjectively perceived probabilities (p) to achieve these goals by means of a particular action.


$$SEU\;\left(A_{i}\right)=\;\sum_{j=1}^{n}p_{ij}*U{\left(O\right)}_{j}$$

Equation 1: Subjective expected utility calculation [29].

According to this model, people’s choices of action are individual and shaped by subjective ideas, and yet can be modelled using a general algorithm that is able to explain why two actors make different decisions in the same situation, e.g., travelling by bike or by car to university.

Additionally, modelling individual decision-making not only helps to understand and explain individual everyday actions, but also to identify entry points for external (political) interventions that might be used to promote behavioural changes.

Fig 3 shows that decision and action are seen as closely linked in this standard model, thus defining away a possible gap between attitudes and behaviour, which almost automatically coincide here.

The SEU concept is a powerful tool for explaining bounded rational behaviour (at the micro level of the individual). Moreover, it is embedded into the broader framework of the “Model of Sociological Explanation” (MSE, cf. [30]), entailing links between the micro and the macro level of societal systems that also help to explain system dynamics (e.g., of transportation systems). Nevertheless, in addressing the *subjective* perception of situational constraints, the standard SEU model underestimates the effects, which these constraints (e.g., living in a city or in the countryside) *objectively* have on travel mode choice (cf. missing arrow in Fig 3 between context and decision). Hence, similar to attitude-related and hybrid choice models, we propose to extend the SEU concept by including those contextual factors, that affect mode choice independently of their subjective perception.

### 2.2. Extended Model of Mobility Behaviour (xMooBe)

In this section, we will develop a model of mobility behaviour, which describes and explains mobility-related choices with a high explanatory power. By combining various strands of research, the model aims to deliver a more complete picture, illustrating the decision-making of different actor types in the case of travel mode choice. By means of matching predicted and actual travel behaviour, this model will also contribute to close the attitude-behaviour gap. Finally, xMooBe helps to identify those factors that shape human mobility behaviour, which can also be taken as starting points for behavioural changes towards sustainable mobility.

The Extended Model of Mobility Behaviour (xMooBe) has been developed and empirically tested in two steps:

**First step: Standard model of utility-maximizing decisions:** As a first step, the sociological model of decision-making (cf. Fig 3), which is based on SEU, has been applied to a dataset from the project InnaMoRuhr (N = 10,782), modelling the presumed decisions of 9,039 respondents (cf. Section 2.4 for more details on the sample). To put it into the terms of TAM: the behavioural intentions have been mathematically calculated, based on (self-reported) preferences and perceptions, instead of relying on (self-reported) explicit statements on intentions. More detailed results of this first empirical test are presented in Section 3.

The explanatory power of the standard model is rather high and matches up to 70 percent of actual mobility behaviour. Nevertheless, the question remains, how to explain the remaining gap: Why do many people who intend to take the car end up using public transport? And why do large numbers of people who prefer the bike use other modes of transportation – a case, where the match is low?

**Fig 4:**
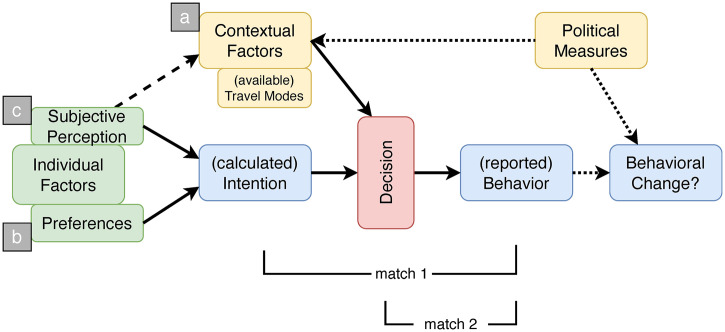
The Extended Model of Mobility Behaviour (xMooBe).

**Second step: Extended model, including contextual factors:** To close this remaining attitude-behaviour gap, the standard model has been further developed into an Extended Model of Mobility Behaviour (xMooBe) by including additional contextual factors such as car ownership or children in the household (see also modified SEU equation in Section 5.1). This extended model, which is shown in Fig 4, aims to explain travel mode choice and to show options for behavioural change.

As described above, intentions in the purely SEU-based model (blue box on the left) result from individual preferences and subjective perceptions (green boxes). Referring to other models, additional contextual factors (yellow box on the left) such as car ownership, distance to work, or availability of public transport services have been considered as part of the decision-making by including them stepwise into the regression analysis (cf. Section 4.2). This way, the explanatory power of the standard model (match 1) can be substantially improved (match 2) regarding the prediction of behaviour (blue box in the middle). Furthermore, entry points for (political) interventions can be identified, which may promote sustainable behavioural changes (blue box on the far right) by shaping the context of individual actions (yellow box on the right). The detailed empirical results of these assumptions are covered in Section 4.

The Extended Model of Mobility Behaviour proposed in this paper is based on the following ideas compared to existing models:

1. It is a deliberately simple model that relies on a manageable number of variables with sufficiently precise impacts on individual decision-making in order to avoid overfitting (i.e., the specification of overly complex models with a myriad of parameters).
2. Instead of relying predominantly on manifest mode-specific preferences and various contextual factors (grey box marked with in Fig 4), xMooBe additionally builds on latent factors. These factors express global, long-term attitudes and preferences of people (b), as well as subjectively perceived probabilities (c) to achieve personal goals through various action alternatives (e.g., available travel modes). This constitutes an important dimension missing in most other models, as reported by Borriello and Rose [13].
3. Instead of referring exclusively to aggregated results of the whole sample (mostly in terms of statistical relations), xMooBe also considers individual decisions and computes the predicted (mobility) behaviour for each person in the data set (pink box in Fig 4). This is based on individual properties, preferences, and situational constraints.
4. By means of this approach, thought experiments can be conducted that help to understand how different actors (or groups of actors) will react to which kind of political measures, promoting sustainable transformation. Finally, this allows to predict if actors will change behaviour or stick with their habits and routines (cf. Section 4.2). This can be further elaborated through simulation experiments with agent-based models (ABM), which also allows investigating the aggregated dynamics (e.g., the diffusion of innovations or modal split) that result from individual choices (cf. [35–37]).

To conclude: xMooBe not only tries to bridge the attitude-behaviour gap, but also to bridge the gap between attitude-related and choice models, similar to hybrid choice models. Utilizing a limited number of subjective (preferences and probabilities) and objective factors (social and infrastructural context), we assume a high degree of match between modelled and actual mobility behaviour as well as a high explanatory power.

Hence, our analysis is guided by the following hypothesis:

(H1) The explanatory power of models of mobility behaviour can be substantially improved by adding contextual factors to the standard sociological model of decision making.

### 2.3. Mixed-methods approach

We use an innovative mixed-methods approach that combines the statistical analysis of survey data, the modelling of (individual) human behaviour via a general algorithm, and experimental procedures. On the one hand, this serves to validate the theoretical concept of xMooBe. On the other hand, these methods are used to identify relevant factors that can be changed in thought experiments. This way, we can investigate options for interventions and to predict the willingness of various actor types to change behaviour in terms of sustainability.

Thought experiments are widely used in natural sciences to predict various outcome, but are rather uncommon in social sciences (except from rare exceptions such as [38]), which are not used to make predictions and to imagine or anticipate future states, based on evidence-related research [39]. Similar or related methodological approaches to predicting human behaviour include, for example, the use of machine learning to identify behavioural or consumption patterns from large amounts of data [40]. Another option for experimentation and predictions on a larger scale are simulation experiments with agent-based models that also cover system dynamics, as documented in [37].

In sum, we use the following methods in our study:

- Statistical analysis of data from an online survey via SPSS, e.g., concerning modal split, preferences etc.;
- Modelling of human behaviour via the SEU algorithm, identifying the best rated mode of transport;
- Evaluation of the attitude-behaviour gap through the ‘non-utilization’ of the best rated mode of transport;
- Binary-logistic regression models to calculate the probability of choosing a mode of transport (car, bike, public transport)
- Thought experiments with fictious people in order to assess the willingness to change behaviour (and the factors triggering this willingness).

This leads to the formulation of our second hypothesis:

(H2) Thought experiments, based on the Extended Model of Mobility Behaviour, give evidence that transport mode choice can be influenced in terms of sustainability by adjusting contextual parameters.

### 2.4. Survey data on current and future mobility

The data for validating xMooBe was taken from a survey conducted as part of the InnaMoRuhr project (“Concept of an integrated, sustainable mobility for the University Alliance Ruhr”). During the lockdown due to the Covid-19 pandemic in Spring 2021, all 130,000 members of three major universities in the Ruhr district (Bochum, Dortmund and Duisburg-Essen) that form the “UA Ruhr” alliance were invited to participate in an online-questionnaire concerning their travel behaviour. All participants were asked to consent to the use of their data. After data cleansing, a total of 10,782 usable data sets remained. It is not a representative sample, compared to the German population, but comprehensive concerning the participation of various groups.

All three universities account for roughly one third of respondents, while women (54.2%) are slightly more represented than men (45.4%). Students constitute the largest group with 7,333 participants (equivalent to 68.0%, cf. Table 1). However, the administrative and technical staff has the highest response rate at 26.7 percent, compared to 18.3 percent of research and teaching staff and only 6.2 percent of students.

**Table 1:** Participation of the three function groups (N = 10,782, source: [41]).

Group	Number	Percent	Response rate
Research & Teaching	1,989	18.4%	18.3%
Administration	1,460	13.5%	26.7%
Students	7,333	68.0%	6.2%

Table 2 shows the UA Ruhr members’ modal split of 2019, i.e., before the outbreak of the Covid-19 pandemic (second column), which is used as a reference to reflect the changes during the lockdown (third column). Additionally, the respondents were asked to state personal wishes regarding their future mobility (third column, for more details see [41]). In all three cases (2019, 2021, future), they were asked to report all trips on an ordinary working day (not only to work, but also for other purposes), including details like used mode of transportation, trip purpose, trip duration, and distance of travel.

**Table 2:** Modal split of UA-Ruhr members based on main modes of transport (source: [41]).

Transport modes	2019	2021 (lockdown)	Future	Diff. 2019/ Future
Public Transport	49.8%	19.5%	36.1%	−13.7 PP
Bus, train etc.	49.1%	18.6%	33.5%	
Sharing, pooling	0.7%	0.9%	2.6%	
Car, Motorbike	31.1%	39.2%	28.2%	−2.9 PP
ICE*	30.2%	37.8%	11.0%	
BEV, FCEV, HEV*	0.9%	1.4%	17.2%	
Bicycle	11.8%	17.5%	27.9%	+16.1 PP
Conventional	10.6%	15.5%	19.9%	
E-Bike, E-Scooter	1.2%	2.0%	8.0%	
Other	7.3%	23.8%	7.7%	+0.4 PP
Walk	7.0%	23.5%	7.4%	
Other	0.3%	0.3%	0.3%	
N=	7,483	6,478	7,766	

*ICE – Internal Combustion Engine; BEV – Battery Electric Vehicle; FCEV – Fuel Cell Electric Vehicle; HEV – Hybrid Electric Vehicle.

The first column “2019” shows a distribution that deviates significantly from the nationwide modal split as documented in “Mobility in Germany (MiD)” [42]: Just about half of university members (49.8% - MiD 10%) used public transport or sharing services, a third (31.1% - MiD 57%) used the car, and only 11.8 percent used bicycles (MiD 11%) as the main mode of transport to get to university.

Mobility patterns of the three function groups differ remarkably, since technical and administrative staff used cars more frequently (33.5 percentage points above average) and public transport less frequently (30.4 percentage points below). Since public transport tickets are included in the study fees in Germany, the proportion of public transport users among students is 12.6 percentage points above the average for all three functional groups (see also S1 Appendix in S1 File).

During the lockdown in 2021 (second column), there was a shift away from public transport (19.5%) towards individual forms of mobility (cars: 39.2%, cycling: 17.5%, walking: 23.5%) as well as new patterns of partial or complete work in the home office [43]. Regarding university members preferred future mobility (column “Future” in Table 2), the passenger car is almost as important as in the past, losing only 2.9 percentage points. Although public transport was able to regain share, it remained a clear loser with a minus of 13.7 percentage points. The highest increase was recorded for the bicycle, which gained 16.1 percentage points and apparently plays an important role in people’s ideas about their future mobility.

Summing up, Table 2 reflects the desire for individual (car/bike), sustainable (electric car, e-bike), and flexible mobility that is not subject to the rigid schemes of classic public transport.

## 3. Results, part 1: Explanation of mobility behaviour through the standard model of action

### 3.1. SEU scores based on survey data

The following calculations apply the method of the standard model (cf. Section 2.1) to the data collected in the InnaMoRuhr project (cf. Section 2.4). Respondents were asked to provide information about their personal preferences (U) related to six goals when travelling. With the help of a slider (from 1 to 10), they were able to indicate how important it is to them to travel fast, cost-effective, environmentally friendly, comfortable, safe, and reliably. In order to provoke trade-offs between conflicting goals, respondents could allocate a total of 30–40 points to the six goals.

As Table 3 shows, reliability was rated highest with 8.1 points on average (column Means) and comfort was rated lowest at 4.7 points. The – partly remarkable – deviations can be seen in the columns “Min” and “Max”. Preference values were used to distinguish five actor types (via cluster analysis), each with distinct features: (1) risk-averse eco-minded, (2) indifferent, (3) pragmatist, (4) comfort-oriented, and (5) cost-conscious eco-minded (cf. [41]).

**Table 3:** Preferences related to six goals and average, mode-specific probabilities (N = 10,782, source: [41]).

Goal	Preferences (U)			Probabilities (p)		
	Min	Means	Max	Car	PT	Bike
Fast	6.2	7.8	8.8	80%	38%	36%
Cost-effective	3.2	6.3	7.2	32%	54%	87%
Environmentally friendly	3.8	5.9	8.0	23%	74%	94%
Comfortable	2.5	4.7	7.5	83%	42%	42%
Safe	3.5	6.2	7.9	68%	65%	44%
Reliably	6.2	8.1	8.9	81%	35%	81%

Respondents were also asked to indicate how likely they thought it was to achieve the six stated goals (from 0 to 100%) by using three modes of transport (car, public transport and cycling). Walking was not considered in order to reduce the length of the questionnaire, and since not all perceptions can be applied in a meaningful way (e.g., perceived reliability).

The values for the probabilities (p) in Table 3 are not particularly surprising, but help to calibrate the SEU algorithm based on the perceptions of the respondents. The variances (not documented in the table) are also considerably smaller than for the U-values. Obviously, there is some consensus that the car is fast, and the bike is environmentally friendly. For the most part, this could be confirmed by a single-factorial analysis of variance (ANOVA) (see S2 Appendix in S1 File).

With the help of the SEU algorithm (cf. Section 2.1) and the survey data, the SEU scores for all three modes of transport were calculated and then the mode of transport with the highest individual utility (SEU score) was determined for each data set (cf. Table 4).

**Table 4:** Top-rated mode of transport based on the SEU algorithm (SEU scores from 0 to 40).

Transport modes	Number	Share	Mean SEU (total)	Mean SEU (top-rated mode only)
Bike	3,037	48.6%	25.0	28.2
Car	2,660	42.6%	24.3	27.8
Public transport	551	8.8%	19.5	27.2
N=	6,248			

As a result, almost half of all respondents (48.6%) rate bicycles as the best mode of transportation, followed by cars (42.6%). Public transport is far behind with only 8.8% of respondents. This is also reflected in the SEU scores (second last column), where the bike with an average value of 25.0 is close ahead of the car (24.3) and far ahead of public transport (19.5). When considering only those respondents who rate the respective mode of transport best (last column), it becomes clear that public transport performs slightly worse even among its supporters (27.2), compared to the other two modes of transport in the groups they prefer (28.2 and 27.8, respectively).

### 3.2. Comparison of modelled and actual behaviour

**Fig 5:**
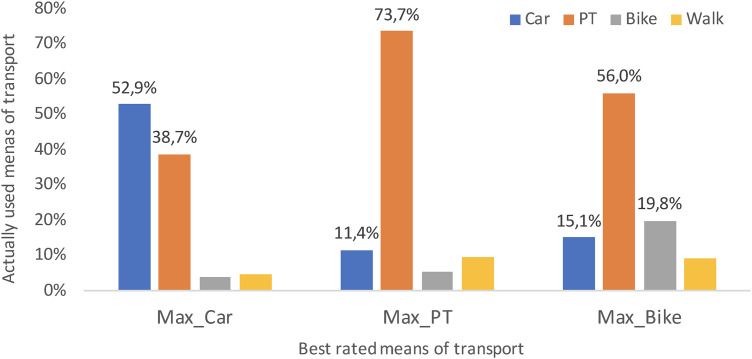
Actually used and highest rated modes of transport (own illustration).

As can be seen in Fig 5, the standard sociological model of mobility behaviour is able to explain actual mode choice with the help of the SEU algorithm – apart from a few deviations: 52.6 percent of those who rate the car highest (Max_Car) actually use it, but 38.9 percent travel to university by public transport. In the case of public transport (Max_PT), almost three-quarters (73.7%) of the (few) people, who rate it highest, actually use it. Both are relatively satisfying values, given the simplicity of the SEU algorithm. However, the discrepancy is greater for bicycles: only just about 20 percent of the (numerous) people who rate the bike highest (Max_Bike) actually use it; however, the majority (56.0%) travel to university by public transport and a small proportion (15.1%) by car.

## 4. Results, part 2: Explanation of mobility behaviour through the extended model of action

These discrepancies between the predicted and actual behaviours, calculated using SEU, can be reduced by including a factor that has not yet been taken into account in the standard sociological model but can be taken over from attitude-related or choice models: the social and spatial context in which the respective person finds themselves. After all, it makes a difference whether they live in the city or in the countryside, whether they have children, whether they own a car, whether there is a well-developed cycling infrastructure, or whether their place of residence is well connected to public transport or not. The quality of the predictions can be increased considerably, if the standard model of mobility behaviour with its two subjective factors is supplemented by another – more objective – factor, the social and infrastructural context.

This inclusion of contextual factors takes place in two steps: First, correlation calculations are used to identify potential factors that prevent a person from actually using their preferred mode of transport. In a second step, these contextual factors are fed into several regression models to check their impact on mobility behaviour. All calculations were carried out using the statistics software SPSS.

### 4.1. Correlation calculations

**Fig 6:**
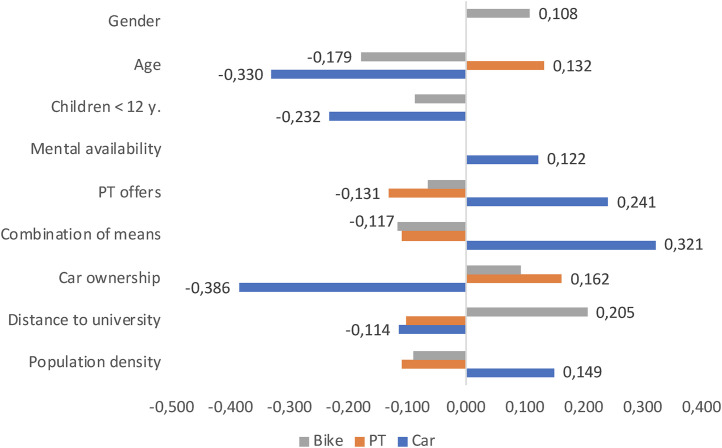
Non-utilization of transportation modes despite highest rating; grey = non-utilization of the bike, blue = non-utilization of the car, green = non-utilization of public transport; Bar not labelled = low correlation (<.100) or only weakly significant (<.05); missing bars = no effect (own illustration).

The results of the correlation calculations can be found in Fig 6. On the x-axis, the negative correlation coefficients (r, according to Spearman) are plotted on the left and the positive correlation coefficients on the right – for the sake of a better overview, only the significant values are reported (p < .001). In each case, the *non-utilization* of a mode of transport despite the best SEU rating (car, public transport, bicycle; 2 levels: 1 = non-use) was correlated with nine contextual factors, most of which were addressed directly and not using Likert scales:

- Gender (2 levels: 0 = male, 1 = female),
- Age (7 levels: 7 = 60 years and older),
- Children under the age of 12 in the household (2 levels: 1 = children in the household),
- The mental availability of eleven innovative alternatives (11 levels: 0 = no alternative mentally available), i.e., the consideration of using sharing services, on-demand shuttles, electric vehicles, and more,
- An additive index, indicating respondents’ evaluation of the local public transport service (5 levels on a Likert scale: 5 = very good rating),
- The habit of using or combining various modes of transport, with higher values indicating a higher degree of sustainability (8 levels: 1 = car only; 4 = car, PT, and bicycle; 8 = none),
- Car ownership (2 levels: 0 = no, 1 = yes),
- The distance to the university (metric scale in kilometres) and
- The population density of the place of residence (metric scale in inhabitants per square kilometre).

#### 4.1.1. Bicycle.

The *non-utilization of the bicycle* despite the best rating (grey bars) is mainly explained by the greater distance to the university (.205, N = 2,515) and by car ownership (.093, N = 2,940). Women also use the bike less often (.108, N = 2,986). Those who do not use the bike are also more likely to be younger (−.179, N = 3,029), are more likely to have no children (−.086, N = 1,066), rate public transport services slightly worse (−.066, N = 2,997), use or combine different modes of transport less often (−.117, N = 1,976) and live in areas with low population density (−.090, N = 2,784), i.e., in the countryside.

#### 4.1.2. Public transport.

The *non-utilization of public transport* despite the best rating (orange bars) is mainly explained by older age (.132, N = 545) and car ownership (.162, N = 495), but also by poorly rated local public transport services (−.131, N = 544). In addition, the short distance to the university (-.102, N = 460) and a low population density (.108, N = 506) play a role. Those who live near the university (in the first case) walk or cycle; those who live in the countryside (in the second case) take the car.

#### 4.1.3. Car.

The *non-utilization of the car* despite the best rating (blue bars) is obviously mainly explained by the lack of car ownership (−.386, N = 2,591), the short distance to the university (−.114, N = 2,057), the lower age (−.330, N = 2,642) and the absence of children in the household (−.232, N = 916). These are probably mainly students who are travelling with the (free) semester ticket but may want to buy their own car with their first child at the latest. In addition, good public transport services (.241, N = 2,603), the habit of using different modes of transport (.323, N = 1,353), and finally the consideration of alternatives (.122, N = 1,886) are factors that explain the non-use of the car.

### 4.2. Regression models

#### 4.2.1. Car use.

The nine factors mentioned above (cf. Fig 5) were adopted into three binary logistic regression models and systematically tested in different combinations to identify the contextual factors that support the use of cars, bicycles, and public transport. The aim was to reduce the mismatch between the utility-calculations based on the standard SEU model and the actual mobility behaviour, reported by respondents of the survey.

All three regression models were developed and tested step-by-step. In the case of the car, the contextual factors were finally reduced to four significant factors (car ownership, age, public transport, and mental availability), which already showed values greater than .200 in the correlation calculations (cf. Section 4.1). All variables included in the model were significant (p < .001) and thus had an impact on the explanatory performance of the model (cf. Table 5). The examination of prerequisites and outliers is described in S4 Appendix in S1 File.

**Table 5:** Binary logistic regression model with the dependent variable “car as main mode of transport” (dummy: 1 = car, 0 = other); values for the different models represent the regression coefficients B; odds ratio Exp (B) in parentheses (own illustration).

Variable	Scaling	Model 0	Model 1	Model 2	Model 3	Model 4	Model 5
Constant		−0.757	−1.741	−6.330	−7.216	−6.099	−5.516
Car rated best	1 = yes 0 = no		1.845 (6.328)	1.637 (5.138)	1.722 (5.595)	1.629 (5.098)	1.616 (5.032)
Car ownership	1 = yes 0 = no			5.145 (171.534)	4.536 (93.312)	4.384 (80.134)	4.257 (70.598)
Age group	1-7				0.408 (1.504)	0.398 (1.489)	0.389 (1.476)
PT index	1-5					−0.317 (0.728)	−0.326 (0.722)
Mental availability	0-10						−0.138 (0.871)
Adj. R^2^ (Nagelkerke)			.214	.398	.451	.472	.487
Chi^2^			1,036.73	2,026.39	2,347.66	2,438.41	1.895.99
Correctly classified		68.1%	71.4%	76.6%	77.6%	78.4%	79.2%
N=		6,307	6,307	6,072	6,044	5,944	4,439

Model 1 already supports the assumption that the exclusive consideration of the subjective utility calculation leads to a moderate explanation of variance (Nagelkerkes R^2^ = .214) as well as a correct overall classification of 71.4 percent of all persons. In the following four models, the classification value gradually rises to 79.2 percent; the quality of the model also improves with the addition of other factors (see also S3 Appendix in S1 File).

Finally, Model 5 has the best values for the quality criteria and – according to the assessments of Backhaus et al. [44] and Field [45] – a good explanation of variance (Nagelkerkes R^2^ = .487, Chi^2^ = 1,895.993; DF = 5; correctly classified overall: 79.2%). In addition to the correct overall classification, the so-called AUC value (“Area under Curve” of the “Receiver Operating Characteristics Curve”) also provides an indicator of the model’s forecast quality (cf. [44]), which is 0.867 for Model 5 and can be classified as “excellent” according to Backhaus et al. (ibid.).

In addition to the regression coefficients, the odds ratios of the various variables are reported (in parentheses in Table 5): These indicate the factor by which the chance increases (> 1) or decreases (< 1) that a person will use a car to travel to university if a characteristic (e.g., age) increases by one step. According to Model 5, the rating of the car (factor 5.032), car ownership (70.598), and age (1.476) are the three factors that increase the probability of using the car, while the evaluation of local public transport services (0.722) and the mental availability of alternatives (0.871) reduce it. This confirms the previous descriptive findings, but now makes it possible to weight the influence of those factors on the use of the car. Car ownership stands out with an odds ratio of 70.598 in Model 5: Owning the car thus increases the probability of using it to drive to university by a factor of 70.

The regression coefficients were also used to calculate the probabilities of car use by two fictitious persons according to the formula proposed by Backhaus et al. [44] and Wentura/Pospeschill [46] (cf. Table 6).

**Table 6:** Probability of car use for two fictitious persons (values in brackets: variable cannot be changed).

Variable	Scale	Person 1	Person 2	Person 1a	Person 1b	Person 1c
Car rated best	1 = yes 0 = no	1	0	1	0	1
Car ownership	1 = yes 0 = no	1	0	(1)	(1)	0
Age group	1-7	6	3	(6)	(6)	(6)
PT index	1-5	1	4	4	4	4
Mental availability	0-10	0	3	3	3	3
**Probability**		**91.4%**	**0.2%**	**72.6%**	**34.5%**	**3.6%**

Person 1 rates the car best (1), owns a car (1), is between 50 and 59 years old (6), rates the public transport services as unsatisfactory (1), and has never been interested in alternatives (0). According to the model, the probability of car use is 95.3 percent.

Person 2 represents an alternative to person 1: S/he rates another mode of transport best instead of the car (0), does not own a car her-/himself (0), is significantly younger at 25–29 years of age (3), rates the public transport services as quite good (4), and has already looked into three alternatives (3). In this case, the probability of car use is 0.2 percent.

In order to persuade person 1 to think about alternatives to the car (and thus turn into person 1a), two contextual factors could be influenced in a thought experiment. This might include improving local public transport services (value increases from 1 to 4) or providing better information, for example via an intermodal mobility app that increases the mental availability of alternatives (value increases from 0 to 3). These measures alone would reduce the likelihood that person 1 would continue to use a car by almost a quarter (from 91.4% to 72.6%). The other factors bracketed in the table (age and car ownership) cannot be influenced by external measures, at least if one rejects the idea that car ownership could be banned.

Further effects could therefore only be achieved (cf. person 1b) if people would get the car completely out of their head – as Weert Canzler [47] and Andreas Knie frequently call for. This could be achieved, if the subjective utility of the car was reduced to such an extent that another mode of transport would be the first choice, e.g., by measures such as a speed limit or a congestion charge for cars. In this case, the probability of car use would drop to 34.5 percent – an option, that is more likely the closer the second-best SEU score is to the (previously) highest value.

Alternatively, the last option (person 1c) would be possible if people could satisfy their mobility needs without their own car due to perfect contextual conditions, e.g., through flexible on-demand services – without the necessity of changing minds. According to this thought experiment, it would be very unlikely (3.6%) that this fictitious person would use the car.

The model, which has been extended by four contextual factors, is thus able to explain actual mobility behaviour in the case of car use with a high degree of accuracy and to largely close the mismatch that results from using only the standard model, i.e., without contextual factors. It also identifies options for initiating a behavioural change towards sustainable mobility, that can be used in further thought or simulation experiments.

#### 4.2.2. Public transport use.

A second regression model for public transport use was developed, which works with the context variables of the car model (car ownership, age, public transport offers, mental availability of alternatives) and additionally includes the distance to the university (cf. Table 7). This model was also reviewed regarding prerequisites, outliers and quality criteria (cf. S5 Appendix in S1 File) and developed in stages; it has satisfactory to good values from mModel 4 onwards.

**Table 7:** Binary logistic regression model with the dependent variable “public transport as main mode of transport” (dummy: 1 = public transport, 0 = other); Variable values for the different models represent the regression coefficients B; odds ratio Exp (B) in parentheses (own illustration).

Variable	Scaling	Model 0	Model 1	Model 2	Model 3	Model 4	Model 5	Model 6
Constant			−0.079	0.657	2.020	1.478	0.795	0.68
PT rated best	1 = yes 0 = no		1.108	1.005	1.035	0.998	0.993	0.952
			(3.029)	(2.731)	(2.814)	(2.713)	(2.698)	(2.591)
Car ownership	1/0			−1.018	−0.853	−0.750	−0.812	−0.841
				(0.32)	(0.426)	(0.472)	(0.444)	(0.431)
Age group	1-7				−0.450	−0.445	−0.476	−0.497
					(0.637)	(0.641)	(0.621)	(0.608)
PT index	1-5					0.152	0.241	0.240
						(1.164)	(1.272)	(1.271)
Mental availability	0-10						0.037	0.075
							(1.079)	(1.078)
Distance university	km							0.038
								(1.039)
Adj. R^2^ (Nagelkerkes)			.029	.085	.193	.200	.239	.251
Chi^2^			137.410	400.762	943.780	967.443	952.977	764.302
Correctly classified		50.3%	53.9%	61.7%	66.3%	66.6%	67.5%	68.9%
N=		6,307	6,307	6,083	6,052	5,952	4,819	3,657

In the following, Model 6 is used, which has the best values in most points: Quality criteria and explanation of variance are not as good as in the car model (see Section 4.2), but overall still satisfactory (Nagelkerkes R^2^ = .251, Chi^2^ = 764.302; df = 6). With the help of the six variables used, the correct classification of the subjects can be increased by almost 20 percentage points – from 50.3 percent (Model 0) to 68.9 percent (Model 6, cf. S3 Appendix in S1 File). With an AUC value of 0.75, the forecast quality of the final model can be classified as “acceptable” [44].

According to Model 6, the evaluation of public transport as the best mode of transport (2.591) as well as the quality of public transport services at home (1.271) have a particularly positive effect on public transport use; in addition, the mental availability of alternatives (1.078) and the distance to the university (1.039 ) have a weakly positive effect. In other words: With each alternative (e-mobility, sharing, etc.), the probability of using public transport increases by about 7.8 percent and by about 3.9 percent with every kilometre of distance.

Car ownership (0.431) and older age (0.608), on the other hand, have a negative effect on public transport use. Again, the regression coefficients (see Table 7) have been used to calculate the probabilities of the use of public transport by two fictitious persons (cf. Table 8).

**Table 8:** Probability of using public transport by two fictitious persons (values in brackets: variable cannot be changed).

Variable	Scaling	Person 1	Person 2	Person 2a	Person 2b	Person 2c
PT rated best	1 = and 0 = no	1	0	0	1	1
Car ownership	1 = and 0 = no	0	1	(1)	(1)	0
Age group	1-7	2	5	(5)	(5)	(5)
PT index	1-5	4	1	4	5	5
Mental availability	0-10	3	0	3	3	3
Distance university	km	10	10	(10)	(10)	(10)
**Probability**		**90.1%**	**11.7%**	**25.3%**	**52.8%**	**72.2%**

Person 1 rates public transport best (1), does not own a car (0), is between 20 and 24 years old (2), has access to a good public transport services at her/his place of residence (4), which is 10 kilometres from the university, and has already dealt with alternatives (3). This results in a probability of using public transport of 90.1 percent, which clearly stands out from person 2 (11.7 percent). This second person forms a contrasting foil insofar as s/he rates a mode of transport another than public transport best (0), owns a car (1), is between 40 and 49 years old (5), has inadequate public transport offers at the place of residence (1), which is also 10 kilometres away, and has never thought about alternatives (0).

Person 2a represents the attempt to attract more type-2 people to public transport and, above all, to change the factors that can be changed with reasonable effort: an attractive public transport offer (value increases from 1 to 4) and improved mental availability, e.g., through advertising campaigns, mobility apps that make alternative suggestions, etc. (value increases from 0 to 3). All other factors, such as car ownership, age, and distance to the university, are impossible or difficult to change (and are therefore kept constant, as indicated by the bracketed values in the table).

As the value of 25.3 percent for person 2a indicates, the probability of using public transport has now doubled (from 11.7% to 25.3%) – a small success, although the probability is still low overall. A greater effect could be achieved (switch to person 2b) if it were possible to provide an optimal public transport offer (5) and also to persuade this person 2b to rate public transport better (value changes from 0 to 1. In this case, the probability would rise to 52.8 percent, which means that, on average, every second journey to the campus by person 2b is made by public transport. Even higher values – see person 2c (72.2%) – could only be achieved if people no longer considered it necessary to own a car because they could satisfy their mobility needs in other ways.

#### 4.2.3. Bicycle use.

Finally, a third regression model for bicycle use was also developed step-by-step. In this case, a combination of the following four contextual factors turned out to be significant: age, car ownership, gender, and distance to university (cf. Table 9).

**Table 9:** Binary logistic regression model with the dependent variable “bicycle as main mode of transport” (dummy: 1 = bike, 0 = other); Variable values for the different models represent the regression coefficients B; odds ratio Exp (B) in parentheses (own illustration).

Variable	Scaling	Model 0	Model 1	Model 2	Model 3	Model 4	Model 5	Model 6
Constant			−3.115	−4.126	−3.843	−3.022	−2.739	−2.677
Bike rated best	1 = yes 0 = no		1.717	1.808	1.793	1.754	1.673	1.571
			(5.569)	(6.101)	(6.007)	(5.778)	(5.329)	(4.809)
Age group	1-7			0.269	0.271	0.343	0.380	0.226
				(1.308)	(1.311)	(1.409)	(1.462)	(1.254)
Gender	1 = woman 0 = man				−0.575	−0.595	−0.597	−0.580
					(0.563)	(0.552)	(0.551)	(0.560)
Distance university	km					−0.092	−0.089	−0.086
						(0.899)	(0.915)	(0.917)
Car ownership	1 = yes 0 = no						−0.532	−0.529
							(0.587)	(0.589)
Function group	1 = employee 0 = student							0.783
								(2.189)
Better cycling network	1 = selected 0 = not sel.							0.443
								(1.557)
Adj. R^2^ (Nagelkerkes)			.117	.147	.160	.249	.262	.280
Chi^2^			390.531	495.144	530.679	714.146	740.712	774.876
Correctly classified		88.3%	88.3%	88.2%	88.2%	87.3%	87.2%	86.6%
N=		6,307	6,307	6,274	6,160	5,006	4,835	4,611

The two variables that had played a central role in the car model (i.e., PT index and mental availability) were not considered here because they did not contribute to a significant improvement in the explanatory content of the bike model. On the one hand, this is plausible, because – unlike in the case of car use or non-use – there is no compelling connection between public transport services and bicycle use and, as has been shown in Section 4.1, the correlation between these two variables was only a very low (r < .1; cf. Fig 6).

On the other hand, this is regrettable insofar as it eliminates two starting points for influencing mobility behaviour in the direction of sustainability, which could be tested in thought experiments like in the two sections before. Additionally, the four factors that finally had proven to be significant in the bicycle model are largely invariant and can hardly be influenced by appropriate measures – at least if one disregards hypothetical and very drastic options, such as a car ban (cf. Table 9).

Despite these limitations, the step-by-step Model 5 already has acceptable values with the four context factors mentioned above (Nagelkerkes R^2^ = .262; correctly classified = 87.2%). Model 1 proves that the exclusive consideration of the subjective utility assessment – unlike in the case of the car – does *not* lead to an acceptable explanation of variance (Nagelkerkes R^2^ = .117) for bicycles. Obviously, there is a large but as yet untapped potential here, to the extent that many people rate bicycles highly but do not use them for various reasons.

To further improve Model 5, the data collected as part of the InnaMoRuhr project were screened again to identify additional, bike-specific contextual factors: the desire for an expansion of the cycle path network to make travelling to campus more sustainable, the desire for access to bike sharing stations, and the ownership of a bike sharing subscription. Additionally, membership of the two function groups, employees or students, also proved to be important.

Model 5 was initially expanded exploratorily to include these four factors; however, only the cycle path network and the functional group contributed significantly to the final Model 6, which was also developed in Table 9 and was used for the following calculations. Analogous to the procedure described above, prerequisites and the presence of outliers were first examined (see S6 Appendix in S1 File).

The results can be seen in Table 9: The positive evaluation of the bicycle (4.809), but also older age (1.254), membership of the functional group of employees (2.189) and the desire for more and better cycle paths (1.557) have a positive effect on the use of the bicycle as the main mode of transport. Unsurprisingly, the distance to the university (0.917) and car ownership (0.589) both have a negative effect. In addition, even if women rate cycling positively, they use this mode of transport less often than men (0.560).

Overall, the extended Model 6 delivers satisfactory results (Chi^2^ = 774.876; df = 7; p < .001; Nagelkerkes R^2^: .280; overall correctly classified: 86.6%; N = 4,611), even if compared to the much better car model (see S3 Appendix in S1 File). In addition, the AUC value for the forecast quality is 0.818 and can therefore be classified as “excellent” [44].

With the extended model, calculations that contrast two fictitious persons also provide meaningful results (cf. Table 10). Person 1 is a male (0) employee (1) between 50 and 59 years of age (6) who lives three kilometres from the university, does not own a car (0), rates the bike best (1) and demands for more and better cycle paths (1). There is a 77.2 percent probability that he will use the bicycle. Person 2 is a female (1) student (0) who is between 20 and 24 years old (2) and must travel 10 kilometres to university. She owns a car (1), rates another mode of transport best (0) and shows no interest in the development of cycle paths (0). In this case, it is rather unlikely (1.5%) that she will cycle to university.

**Table 10:** Probability of bicycle use by two fictitious persons (values in brackets: variable cannot be changed).

Variable	Scaling	Person 1	Person 2	Person 2a	Person 2b	Person 2c	Person 2d
Bike rated best	1 = yes 0 = no	1	0	0	1	1	1
Age groups	1-7	6	2	(2)	(2)	(2)	(2)
Gender	1 = woman 0 = man	0	1	(1)	(1)	(1)	(1)
Distance university	km	3	10	(10)	(10)	(10)	5
Car ownership	1/0	0	1	(1)	(1)	0	0
Function group	1 = employee 0 = student	1	0	0	0	0	0
Cycling network	1 = selected 0 = not sel.	1	0	1	1	1	1
**Probability**		**77.2%**	**1.5%**	**2.3%**	**19.8%**	**29.6%**	**39.2%**

This does not change significantly if you can interest her in the development of cycle paths (person 2a: 2.3%) – unfortunately the only context variable that is available as an „adjusting screw“ in the bike model. Only when she changes her assessment of the three modes of transport in favour of the bike, the probability of using the bike increases to almost 20 percent (person 2b). If it were possible to satisfy her mobility needs without owning a private car, this figure would rise to almost 30 percent (person 2c), which conversely means that this person is seventy percent likely to use public transport or car sharing, but not the bicycle. Even moving closer to the university (person 2d) only increases this figure to just under 40 percent.

As a conclusion, it can be stated that the bike model also produces good and plausible results but suffers from the fact that it contains too few bike-specific contextual factors that could help to close the delta between modelled and real mobility behaviour. This points to gaps in the (survey) data that cannot be closed retrospectively. For example, the questionnaire did not ask about factors such as weather, health, condition of cycle paths, etc., which might influence the willingness to cycle.

## 5. Conclusion/Discussion: Modelling behavioural change

In conclusion, a strategy of combining various approaches and, simultaneously, reducing the number of factors leads to a sociological model of mobility behaviour, that yields good to very good results when trying to explain everyday human behaviour. Like hybrid choice models, xMooBe integrates dimensions from attitude-related and choice models, but puts stronger emphasis on mode-unspecific, latent preferences (cf. also [13]) as well as on subjectively perceived probabilities of achieving individual goals by various means (of transport) – a dimension, missing in most other models.

### 5.1. Dynamics of complex social systems

xMooBe is an attempt to further develop and to validate the sociological theory of action, which itself is part of a larger endeavour of explaining the dynamics of complex social systems, characterized by the interaction of actors (micro level) and the system (macro level). Agent-based models (ABM) that investigate the sustainable transformation of complex socio-technical systems such as urban transportation, need a valid model of human action at the micro level to generate meaningful results at the aggregate (macro) system level [36].

As could be shown in Section 3, the standard model of decision-making, rooted in analytical sociology, is a good starting point, but it should be supplemented by a third factor that has so far received little attention: the social and infrastructural context, i.e., a rather objective variable that complements the two subjective factors of the standard model (individual preferences and subjective perception of the situation). Consequently, the SEU formula must be modified accordingly, whereby it is assumed that the contextual factors (*Cf*) mainly influence subjective perception (cf. Equation 2). In this way, a very high level of agreement between modelled and real behaviour can be achieved – with acceptable to excellent explanatory power and correct classification values of 70 to almost 80 percent.

Hence, hypotheses H1 can be confirmed:

(H1) The explanatory power of models of mobility behaviour can be substantially improved by adding contextual factors to the standard sociological model of decision making.


$$SEU\;\left(A_{i}\right)=\;\sum_{j=1}^{n}{\left(p*Cf\right)}_{ij}*U{\left(O\right)}_{j}$$

Equation 2: Modified SEU formula for utility calculation

According to the extended Model of Mobility Behaviour (xMooBe; cf. Fig 4 in Section 2.2) mobility behaviour is thus characterized by (a) the social and infrastructural context (place of residence, children at home, car ownership, etc.), (b) the individual preferences, and (c) the subjective perception of the situation in which one finds oneself (e.g., the available mobility offers), which may differ from the objective situation.

### 5.2. Bridging the attitude-behaviour gap

In many ways, our approach and findings are consistent with those of other researchers, such as Kroesen et al. who calculated three different models for car, public transport and bicycle use and found varying degrees of dissonance between attitudes and transport use – the “highest for the bicycle followed by public transport and then the car” [14]. However, we would refrain from calling this phenomenon “cognitive dissonance” [12] since the mismatch is rooted in the discrepancy between desire and reality, and not in people’s brains.

However, with the help of the extended model xMooBe, the attitude-behaviour gap can be at least partially bridged, for example by answering the question of why so many people who rate the bicycle best still use the car or public transport. The answer is not too complicated: It is the additional contextual factors such as children in the house, distance to university or owning a car that prevent people from doing what they would like to do in an ideal world, or in terms of TPB: what is their behavioural intention.

xMooBe helps to better understand and to explain everyday decisions of heterogeneous individuals, that might be willing to change behaviour in case of (a) changing contextual conditions, in case of (b) changing preferences (“Wertewandel”, e.g., towards sustainability), or in case of (c) changing perceptions of the world (cf. Fig 4 in Section 2.2). xMooBe makes it possible to calculate and thus explain everyday decisions and clarify options for behavioural change by identifying relevant factors and their interrelationships. Above all, the model helps to better understand why self-reported behavioural intentions do not always match actual behaviour.

Thought experiments, conducted in Section 4.2, give evidence that hypothesis H2 can also be confirmed:

(H2) Thought experiments, based on the Extended Model of Mobility Behaviour give evidence that transport mode choice can be influenced in terms of sustainability by adjusting contextual parameters.

### 5.3. Policy recommendations

Hence, these empirically tested theoretical considerations have implications for any attempt to promote and advance mobility transitions. After all, it is not only important to develop new mobility services, but also to provide appropriate information and thus influence and shape subjective perceptions. However, information on new mobility options or services should not be scattered according to the ‘watering can’ principle, but should be targeted at individual people and their subjective view of things. Most researchers make policy recommendations that emphasize the need of changing individual attitudes. However, attitudes change very slowly, whereas the contextual conditions, such as new cycle paths, can change more quickly. In addition, subjective perceptions can be influenced more easily and changed more quickly, e.g., through innovative mobility apps that provide customised offers based on people’s individual needs. Simulation experiments with ABM might be a useful tool to test these assertions and to provide policy makers with evidence on various scenarios of future mobility.

### 5.4. Critical discussion and outlook on future studies

Nevertheless, the models presented here have limitations. First, concerning the bike model, further (externally) influenceable contextual factors should be considered. Unfortunately, the survey was not designed to capture these factors as it did in the case of the car and public transport. Second, it is advisable, to map not only the effects of policy measures (e.g., improving public transport) on individual persons, but also on larger populations, in order to cover the non-linear effects of uncoordinated actions of various people, affecting each other (as in the case of congestion). The conduct of thought experiments is a first step to better understand everyday mobility behaviour. However, xMooBe could also be applied in the form of agent-based simulations to test its assumptions by means of simulation experiments (cf. [48]).

In addition, the xMooBe model should be validated in future studies with further data from other sources, which would help to substantiate the claim that it has a high explanatory power. This could also help to address the generalisability issue, as xMooBe was developed using data from a university population that does not represent the entire population. Finally, the regression models presented here assume separate individual decisions in the choice of modes of transport (“Do I take the bicycle today or not? Do I take the car today or not?“), although cross-modal factors are taken into account in each case (e.g., car ownership, public transport services). Consequently, the model could be refined in future studies by applying a multinominal logistic regression to better reflect direct competition between modes of transport (“Do I use bicycles, cars or public transport today?“).

## Supporting information

S1 File S1 Appendix. Comparison of preferences by function groups (ANOVA). S2 Appendix. Comparison of probabilities by actor types (ANOVA). S3 Appendix. Correct overall classification. S4 Appendix. Examination of prerequisites and outliers (car model). S5 Appendix. Examination of prerequisites and outliers (public transport model). S6 Appendix. Examination of prerequisites and outliers (bicycle model). Appendices S4 to S6 refer to recommendations by [5,44,45,49,50]
(ZIP)
