# Supplementary material for: Bridging the attitude-behaviour gap: An explanation of travel mode choice using analytical sociology
Source: PLoS One. 2025 Oct 15;20(10):e0330073. doi: 10.1371/journal.pone.0330073 (PMC12527145; doi:10.1371/journal.pone.0330073)
Supplement: S1 File — S1 Appendix. Comparison of preferences by function groups (ANOVA). S2 Appendix. Comparison of probabilities by actor types (ANOVA). S3 Appendix. Correct overall classification. S4 Appendix. Examination of prerequisites and outliers (car model). S5 Appendix. Examination of prerequisites and outliers (public transport model). S6 Appendix. Examination of prerequisites and outliers (bicycle model). Appendices S4 to S6 refer to recommendations by [5,44,45,49,50] (ZIP) [file pone.0330073.s001.zip › S1 Appendix. Comparison of preferences by function groups.docx]

## **Appendix 1: Comparison of preferences by function groups (ANOVA)**

The high proportion of students in the InnaMoRuhr sample (68.0 %) might cause concerns of distorting the analysis. Surprisingly, differences only can be observed in mobility patterns (which is the dependent variable to be explained by analysis) and in various contextual factors as age or children at home (which is one major factor in the model, explaining different out-comes), but not in the preferences and perceptions (cf. Section 3.1) of the three groups considered. As an ANOVA has shown, there is only one minor difference concerning the preference for cost-effective travelling (eta2: .041 [low, when comparing all three groups] and also .041 [low, when comparing employees and students]). Additionally, public transport is perceived slightly more as cost-effective by students (eta2: .060 and .053 – both medium), probably because it is free of charge due to the semester ticket. Hence, the authors decided to use the whole data sample and to explore different mobility patterns of all three groups.
