# Supplementary material for: Bridging the attitude-behaviour gap: An explanation of travel mode choice using analytical sociology
Source: PLoS One. 2025 Oct 15;20(10):e0330073. doi: 10.1371/journal.pone.0330073 (PMC12527145; doi:10.1371/journal.pone.0330073)
Supplement: S1 File — S1 Appendix. Comparison of preferences by function groups (ANOVA). S2 Appendix. Comparison of probabilities by actor types (ANOVA). S3 Appendix. Correct overall classification. S4 Appendix. Examination of prerequisites and outliers (car model). S5 Appendix. Examination of prerequisites and outliers (public transport model). S6 Appendix. Examination of prerequisites and outliers (bicycle model). Appendices S4 to S6 refer to recommendations by [5,44,45,49,50] (ZIP) [file pone.0330073.s001.zip › S2 Appendix. Comparison of probabilities by actor types.docx]

## **Appendix 2: Comparison of probabilities by actor types (ANOVA)**

Although the 18 perceived probabilities of goal achievement (6 dimensions for each of the three modes of transport) differ significantly between the five types of actors (highest p < .014), only one case (perceived comfort of public transport) has a medium effect (ω2 > ,06; cf. 45) – the other assessments differ only slightly (mostly omega-squared of approximately .03).

The standard deviations of all perceived probabilities amount to 22 percentage points. This means, for example, that the perceived probability of getting to university quickly by public transport deviates by 22 percentage points from the average rating of 38 percent (cf. Table 3).
