# Supplementary material for: Bridging the attitude-behaviour gap: An explanation of travel mode choice using analytical sociology
Source: PLoS One. 2025 Oct 15;20(10):e0330073. doi: 10.1371/journal.pone.0330073 (PMC12527145; doi:10.1371/journal.pone.0330073)
Supplement: S1 File — S1 Appendix. Comparison of preferences by function groups (ANOVA). S2 Appendix. Comparison of probabilities by actor types (ANOVA). S3 Appendix. Correct overall classification. S4 Appendix. Examination of prerequisites and outliers (car model). S5 Appendix. Examination of prerequisites and outliers (public transport model). S6 Appendix. Examination of prerequisites and outliers (bicycle model). Appendices S4 to S6 refer to recommendations by [5,44,45,49,50] (ZIP) [file pone.0330073.s001.zip › S3 Appendix. Correct overall classification.docx]

## **Appendix 3: Correct overall classification**

The weighted averages of the correct overall classification (second row) result from the correct classification of use (third row) and non-use (fourth row) of respective mode of transport.

|  | Car model | PT model | Bicycle model |
| --- | --- | --- | --- |
| Weighted average | 79.2 | 68.9 | 86.6 |
| Use (sensitivity) | 64.5 | 71.8 | 14.6 |
| Non-Use (specifity) | 86.1 | 66.2 | 98.2 |

Table 11: Correct overall classification of the three models in percent (%)

Regarding the bicycle model, sensitivity (bike use correctly classified) could be increased at the expense of specificity (bike non-use correctly classified) by choosing a lower threshold for the correct classification of bicycle use (e.g. a cut value of 20 % instead of 50 %). However, this would not change the quality of the forecast in terms of the AUC value (cf. “Bicycle use” in Section 4.2).
