# Supplementary material for: Bridging the attitude-behaviour gap: An explanation of travel mode choice using analytical sociology
Source: PLoS One. 2025 Oct 15;20(10):e0330073. doi: 10.1371/journal.pone.0330073 (PMC12527145; doi:10.1371/journal.pone.0330073)
Supplement: S1 File — S1 Appendix. Comparison of preferences by function groups (ANOVA). S2 Appendix. Comparison of probabilities by actor types (ANOVA). S3 Appendix. Correct overall classification. S4 Appendix. Examination of prerequisites and outliers (car model). S5 Appendix. Examination of prerequisites and outliers (public transport model). S6 Appendix. Examination of prerequisites and outliers (bicycle model). Appendices S4 to S6 refer to recommendations by [5,44,45,49,50] (ZIP) [file pone.0330073.s001.zip › S4 Appendix. Examination of prerequisites and outliers (car model).docx]

## **Appendix 4:** **Examination of prerequisites and outliers (car model)**

According to the Box-Tidvell method, the requirement of linearity is fulfilled for all metric variables except the distance to the university.

The correlations between the individual factors were low (highest r = .20), the variance inflation factors (VIF) were below 10 or, on average, not significantly greater than 1 (highest VIF = 1.11), and the tolerance values were above the critical threshold of 0.2 (smallest tolerance value = 0.88). Therefore, it can also be assumed that there is no multicollinearity (cf. 44, 45).

In all models, outliers were identified based on Field’s recommendations (45) and excluded from further analyses. Cases with strikingly high studentized residuals (>= ± 3) and excessively high influence on the model (Cooke distance > 1 or leverage > 0.2) were excluded; cf. (45) and (5, 49, 50). There were 6 (Model 5), 8 (Models 4 and 3) and 11 (Model 2) cases, respectively.
