# Supplementary material for: Bridging the attitude-behaviour gap: An explanation of travel mode choice using analytical sociology
Source: PLoS One. 2025 Oct 15;20(10):e0330073. doi: 10.1371/journal.pone.0330073 (PMC12527145; doi:10.1371/journal.pone.0330073)
Supplement: S1 File — S1 Appendix. Comparison of preferences by function groups (ANOVA). S2 Appendix. Comparison of probabilities by actor types (ANOVA). S3 Appendix. Correct overall classification. S4 Appendix. Examination of prerequisites and outliers (car model). S5 Appendix. Examination of prerequisites and outliers (public transport model). S6 Appendix. Examination of prerequisites and outliers (bicycle model). Appendices S4 to S6 refer to recommendations by [5,44,45,49,50] (ZIP) [file pone.0330073.s001.zip › S5 Appendix. Examination of prerequisites and outliers (public transport model).docx]

## **Appendix 5:** **Examination of prerequisites and outliers (public transport model)**

There was no multicollinearity (highest coefficient r in the correlation matrix = 0.284 between distance and public transport service evaluation; largest VIF = 1.14; smallest tolerance value = 0.88).

According to the Box-Tidvell method, there was no linearity in the public transport service evaluation. Therefore, this variable was converted into a dummy variable as a test (1 = satisfactory service available, 0 = no satisfactory service) and the regression was repeated. The effect of this recoded, binary variable was still significant (B = .792; Exp (B) = 2.208; p < .001) and there were hardly any changes to the overall model (e.g. AUC = .750; R2 = .254; correctly classified overall = 68.7), which is why, for reasons of uniformity (cf. car model in Section 4.2), we decided to retain the original variable with five characteristics.

Furthermore, according to the recommendations of Field (45), no outliers could be identified (cf. Appendix 4).
