# Supplementary material for: Bridging the attitude-behaviour gap: An explanation of travel mode choice using analytical sociology
Source: PLoS One. 2025 Oct 15;20(10):e0330073. doi: 10.1371/journal.pone.0330073 (PMC12527145; doi:10.1371/journal.pone.0330073)
Supplement: S1 File — S1 Appendix. Comparison of preferences by function groups (ANOVA). S2 Appendix. Comparison of probabilities by actor types (ANOVA). S3 Appendix. Correct overall classification. S4 Appendix. Examination of prerequisites and outliers (car model). S5 Appendix. Examination of prerequisites and outliers (public transport model). S6 Appendix. Examination of prerequisites and outliers (bicycle model). Appendices S4 to S6 refer to recommendations by [5,44,45,49,50] (ZIP) [file pone.0330073.s001.zip › S6 Appendix. Examination of prerequisites and outliers (bicycle model).docx]

## **Appendix 6:** **Examination of prerequisites and outliers (bicycle model)**

The prerequisites of linearity and non-multicollinearity were also fulfilled in the bicycle model (largest VIF = 1.97; smallest tolerance value = 0.51): Although there was a high correlation between age and the functional group (r = -0.644) – according to the rules of thumb listed by Field, only values from ±0.8 are to be considered critical for non-multicollinearity (45, p. 534).

Analogous to the outlier identification described in Appendix 4, only 6 cases were affected in models 4 and 5; in the extended Model 6 there were 5 outliers.
